# Uncommon Bedfellows: Coexistent T-Cell Large Granular Lymphocytic Leukaemia and Hodgkin Lymphoma—A Case Report

**DOI:** 10.1155/crh/6159755

**Published:** 2025-11-10

**Authors:** Daniel van Tonder, Brendon Roets, Nicole Holland

**Affiliations:** ^1^Department of Haematopathology, Lancet Laboratories, Johannesburg, South Africa; ^2^Human Anatomy and Physiology, University of Johannesburg, Johannesburg, South Africa

**Keywords:** Hodgkin lymphoma, lymphocytosis and case report, relapse, T-cell large granular lymphocytic leukaemia

## Abstract

This case describes an exceptionally rare co-occurrence of Hodgkin lymphoma (HL) and T-cell large granular lymphocytic leukaemia (T-LGLL), highlighting the diagnostic and therapeutic complexity of dual lymphoid neoplasms. A 39-year-old African man presented with B symptoms and was diagnosed with stage IIIB HL and achieved remission following six cycles of doxorubicin (adriamycin), bleomycin, vinblastine and dacarbazine (ABVD) chemotherapy with external beam radiotherapy. At diagnosis, bone marrow evaluation revealed lymphocytosis with aberrant T-cell phenotypes and biclonality in the T-cell receptor rearrangements, suggestive of a coexistent clonal T-cell process. Following treatment, he developed persistent lymphocytosis with conversion to a monoclonal T-cell population, indicating clonal selection. Nearly 3 years later, the patient relapsed with HL, accompanied by bone marrow infiltration by large granular lymphocytes with a monoclonal, aberrant cytotoxic T-cell phenotype, consistent with T-LGLL. This case is notable for the evolution of T-LGLL in the context of relapsed HL, possibly due to therapy related selection of a pre-existing T-cell clone. Literature on the coexistence of HL and T-LGLL is sparse, underscoring the rarity of this presentation.

## 1. Introduction

Hodgkin lymphoma (HL) is a haematological malignancy of a germinal centre B-lymphocyte, characterized by the presence of Reed–Sternberg cells within an inflammatory background and CD15/CD30 positivity [1]. Chemotherapy and radiotherapy ensure high cure rates, with a 5-year overall survival rate exceeding 90%. Despite these advances, approximately 25% of the patients will experience a relapse, posing significant clinical challenges [2]. T-cell large granular lymphocytic leukaemia (T-LGLL) is a rare heterogeneous disorder, accounting for 2%–6% of chronic lymphoproliferative cases, which is characterized by persistent clonal expansion of cytotoxic T-cells with no discernable cause [1, 3]. The latest World Health Organization (WHO) criteria for diagnosing T-LGLL require either all three essential criteria or two essential criteria and one desirable criterion to be satisfied. The essential criteria include an increase in circulating cytotoxic T-cells, evidence of T-cell monoclonality and the presence of an aberrant T-cell phenotype, typically CD8 positive with downregulated CD5 and or CD7 and abnormal expression of CD16 and NK-cell receptors. The desirable criteria include demonstration of intrasinusoidal cytotoxic lymphocyte infiltrates in the bone marrow by immunohistochemistry (IHC) or the demonstration of *STAT3 or STAT5B* mutations [4, 5]. The pathophysiology remains unclear. However, it is suggested the clonal lymphocytosis is caused by prolonged antigenic stimulation, leading to apoptotic dysregulation within the *JAK/STAT* pathway [6]. The condition is associated with autoimmune conditions and haematological neoplasms, most frequently plasma cell dyscrasias, chronic lymphocytic leukaemia, B-cell non-HL and myelodysplastic syndrome [7, 8]. A review of the literature reveals a scarcity of cases describing the association between HL and T-LGLL. Furthermore, to the best of our knowledge this is the first case report describing chemotherapy-induced selection of a coexistent T-cell clone, with the consequent emergence of T-LGLL in a case of relapsing HL. Therefore, the aim of this case report is to highlight the presentation and diagnosis of such a case.

## 2. Case Report

A 39-year-old African man, with no known medical comorbidities, presented with a 1-month history of right sided cervical lymphadenopathy and B symptoms in November 2021. A diagnosis of HL was confirmed on the histology of an excisional lymph node biopsy. He was diagnosed with stage III BX disease based on clinical and radiological investigations. Computed tomography (CT) scan revealed disease on both sides of the diaphragm with splenic involvement. The full blood count (FBC) revealed a haemoglobin of 11.5 g/dL, white cell count of 3.66 × 10^9^/L, platelet count of 223 × 10^9^/L, absolute lymphocyte count of 2.9 × 10^9^/L and an absolute neutrophil count of 0.62 × 10^9^/L. A staging bone marrow biopsy revealed no bone marrow involvement by the HL. However, a marrow lymphocytosis of 28% was noted. Flow cytometry on bone marrow aspirate revealed a population of 46% T-cells with expression of CD3, CD2 and CD5. Half of these cells showed aberrant loss of CD7, as well as loss of CD4/CD8 or dim CD8 expression. T-cell receptor gamma gene (TCRG) clonality revealed a biclonal T-cell receptor (IdentiClone TCRG Gene Clonality Assay).

**Figure 1:**
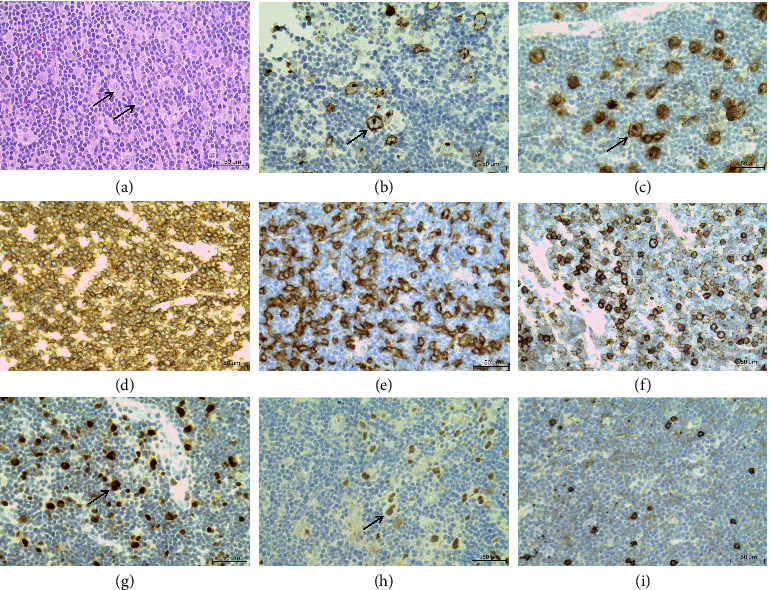
Lymph node biopsy. (a) Haematoxylin and eosin staining showed Reed–Sternberg cells (black arrow) in a polymorphous inflammatory infiltrate, immunohistochemistry showed (b) CD15 positive membranous and Golgi staining in Hodgkin cells (black arrow), (c) CD30 positive membranous and Golgi staining in Hodgkin cells (black arrow), (d) CD3 positive staining showed a large number of background T-lymphocytes, (e) predominantly CD4 positive T-cells, (f) CD8 positive T-cells, (g) MUM-1 positive nuclear staining in Hodgkin cells (black arrow), (h) PAX-5 weakly positive in Hodgkin cells (black arrow) and (i) CD20 negative in Hodgkin cells and highlights background B-cells. Micrographs were produced using the Leica ICC50 HD microscope, magnification of × 50, scale bar is 50 μm.

He achieved remission of his HL following six cycles of doxorubicin (adriamycin), bleomycin, vinblastine and dacarbazine (ABVD) and external beam radiation therapy (EBRT). Following chemoradiation, the FBC showed improvement with a haemoglobin of 14.1 g/dL, white cell count of 13.99 × 10^9^/L, platelet count of 223 × 10^9^/L, absolute lymphocyte count of 10.07 × 10^9^/L and an absolute neutrophil count of 2.80 × 10^9^/L. Repeat flow cytometry on peripheral blood revealed a population of 68% circulating T-cells with a similar aberrant immunophenotype to that previously described. However, the repeat TCRG clonality revealed monoclonal TCR expression. The periheral blood lymphocytosis was noted post-treatment and persisted during the follow up visits in 2023 and 2024, with no further flow cytometry or TCRG clonality performed. In August 2024, he had a recurrence of bilateral cervical lymphadenopathy and B symptoms. Repeat biopsy of a right cervical lymph node (Figure 1) showed prominent lacunar- and classical Reed–Sternberg cells in a background of polymorphous inflammatory infiltrate. Immunohistochemical staining of the tumour cells showed strong membranous and Golgi staining for CD15 and CD30, nuclear positive staining for multiple myeloma oncogene 1 (MUM-1) and weak staining for paired box protein 5 (PAX-5). The tumour cells were negative for CD20. Positive CD3 staining showed numerous background T lymphocytes, which were predominantly CD4 positive.

Positron emission tomography–computed tomography (PET-CT) scan confirmed nodal disease above and below the diaphragm with increased 18-fluorodeoxyglucose (FDG) uptake involving the left level IIb node (inferior C3-C4 level) 21 × 12 mm (SUV 5.24), left level IIb node (superior C2-C3 level) 20 × 11 mm (SUV 4.20), right level IVa node (medial) 25 × 16 mm (SUV 3.71), left superior mediastinal node 18 × 14 mm (SUV 3.40), right hilar node 26 × 21 mm (SUV 3.20), pulmonary nodule in the left lower lobe posterior segment 13 × 11 mm (SUV 4.38), multiple enlarged abdominal and pelvic lymph nodes (SUV 2.93) and spleen with a vertical length of 16.7 cm.

**Figure 2:**
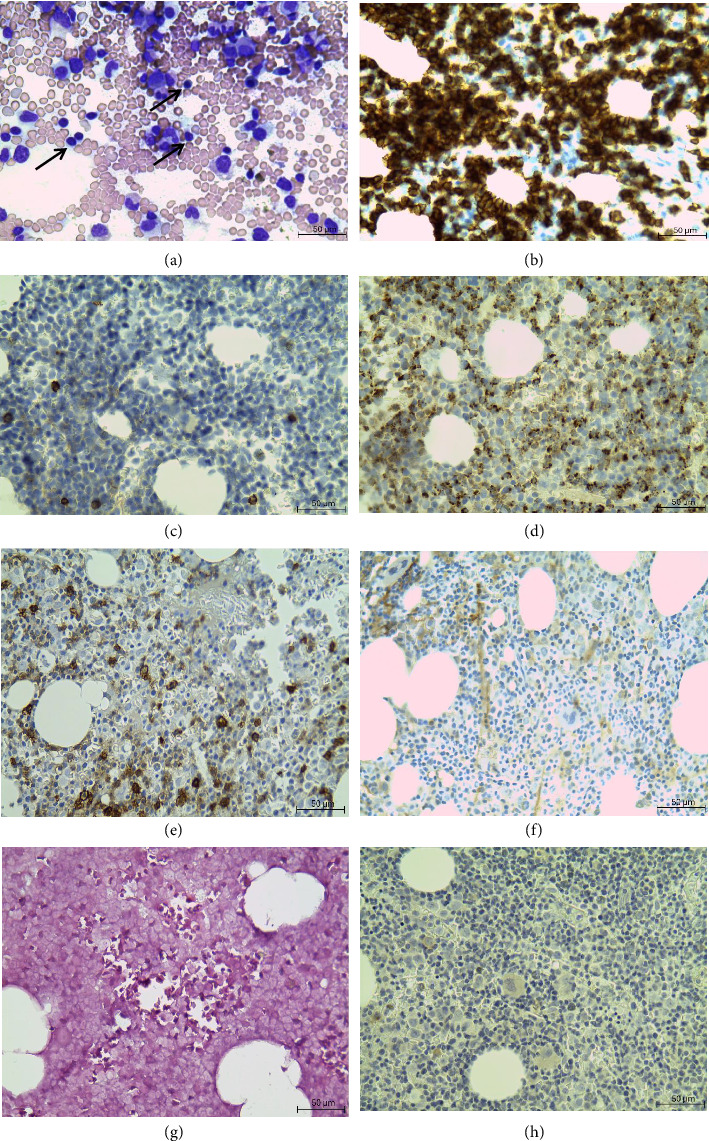
Bone marrow biopsy. (a) May–Grünwald and Giemsa-stained aspirate showing cellular trails with lymphocytosis (black arrows). Immunohistochemistry showed (b) CD3 positive T-cell infiltration, (c) occasional CD20 positive B-cells, (d) TIA-1 positive clusters highlighting cytotoxic T-cell infiltration, (e) predominantly CD8 positive T-cells, (f) scattered CD4 positive T-cells, (g) EBER negativity and (h) CD30 negativity highlighting the absence of Hodgkin cells. Micrographs were produced using the Leica ICC50 HD microscope, magnification of × 50, scale bar is 50 μm.

His FBC showed a mild normochromic anaemia (haemoglobin of 12.6 g/dL), with an elevated white cell count of 17.54 × 10^9^/L with a lymphocytosis of 15.52 × 10^9^/L and mild neutropenia of 1.36 × 10^9^/L and platelet count of 189 × 10^9^/L. Review of the peripheral smear showed an expanded lymphocyte population with small mature forms and large granular forms. Bone marrow aspirate (Figure 2) and trephine biopsy showed hypercellular particles with reduced granulopoiesis and erythropoiesis, owing to bone marrow infiltration by mature lymphoid cells (53% on the aspirate). These cells were small with scanty cytoplasm and a clumped chromatin pattern. The infiltrate was CD3+ and CD20-. Furthermore, large clusters of TIA-1 and CD8 positive cells were observed. Ebstein–Barr virus-encoded small RNAs (EBER) was negative, and CD30 did not highlight the presence of any Reed–Sternberg cells.

**Figure 3:**
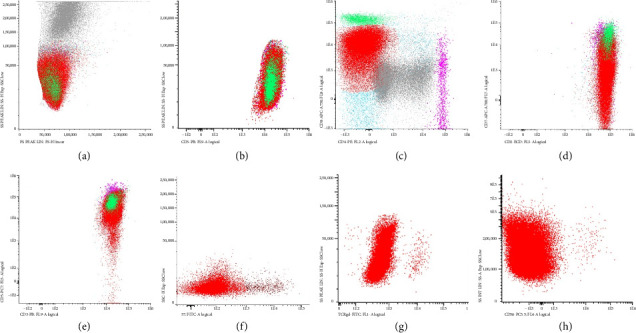
Flow cytometry. (a) White cell distribution, (b) CD3 positive T-cells, (c) 88% of CD3 T-cells express dim CD8 (red population), 2% express bright CD8 (green population) and 1.7% express bright CD4 (pink population), (d) dim CD8 population shows a range of CD7 expression with absent expression in 50% of cells, (e) CD5 expression is normal in most cells, with absent expression in 3% of cells, (f) no significant CD57 expression, (g) no significant TRC gamma–delta expression and (h) no significant CD56 expression. A minimum of 100,000 events were acquired and populations gated on CD45 positive events.

Flow cytometry (Figure 3) on a bone marrow sample revealed that T-cells comprised 32% of the cells analysed. A significant subpopulation (28% of the total sample and 88% of the T-cell population) expressed CD3++, dimmer CD8, CD2++, CD5++ and a range of CD7, with no expression of CD56, CD16, CD57, CD25, CD26 or gamma delta T-cell receptor. There was no overt evidence of a B-cell lymphoproliferative disorder. T cell receptor gamma gene (TCRG) clonality was assessed using multiplex polymerase chain reaction (Invivoscribe TCR Gene Clonality Assay) and once again showed a monoclonal pattern. No somatic mutations including *STAT3* mutations were detected by lymphoid DNA next generation sequencing (NGS). These findings were consistent with T-LGLL in the setting of relapsed HL.

## 3. Discussion

This case highlights an exceptionally rare coexistence of HL and T-LGLL, emphasizing the diagnostic and therapeutic challenges when distinct lymphoid neoplasms arise in the same patient. The key feature was the evolution from an initially biclonal T-cell population to a persistent monoclonal cytotoxic T-cell clone following successful HL treatment, suggesting an underlying lymphoproliferative process that was unmasked or selected by therapy.

At disease onset, bone marrow examination demonstrated no HL involvement but did reveal a marrow lymphocytosis with abnormal T-cell phenotypes and biclonal T-cell receptor expression, indicating the coexistence or pre-existence of an evolving T-cell lymphoproliferative process, although not initially recognized as neoplastic. The original workup was performed at a different centre, influencing diagnostic procedures followed and the diagnosis of T-LGLL was not made at that point; however, it is possible that the significant neutropenia documented at disease presentation was related to the underlying T-cell lymphoproliferative disorder. The later emergence of a dominant monoclonal T-cell population raises the possibility that chemoradiation exerted selective pressure, disrupting immune homeostasis and promoting clonal expansion of the pre-existing cytotoxic T-cells. Similar post-treatment clonal expansions have been described in contexts of chronic immune stimulation and marrow suppression, where dysregulated cytokine signalling or reduced immune surveillance favours persistence of an aberrant clone [9]. However, the current observations are limited to a single case and should be further investigated.

Nearly 3 years after the initial diagnosis, the patient presented to another centre with recurrent B symptoms and lymphadenopathy. A relapse of HL was confirmed by cervical lymph node biopsy, characterized by typical Reed–Sternberg cells in an inflammatory background with CD15/30 positivity. Additionally, the FBC showed worsening lymphocytosis, mild anaemia and neutropenia. Peripheral smear and bone marrow studies performed at our institution demonstrated lymphoid infiltration by mature small and large granular lymphocytes, confirmed immunophenotypically as aberrant cytotoxic T-cells with CD3+, CD2+, CD5+, dim CD8 and variable CD7 expression, with absence of CD56, CD16 and CD57. T-Cell receptor gene monoclonality was confirmed using polymerase chain reaction (PCR). The findings satisfied the current WHO criteria for T-LGLL diagnosis. Interestingly, the immunophenotypic expression in this case was different to that commonly described. The typical immunophenotypic expression of T-LGLL reported is: CD2+ CD3+, CD7+, CD8+ and αβ T-cell lineage with CD57 and CD16 expressed in most cases [4, 5]. The atypical phenotype is our case may reflect functional heterogeneity within T-LGLL or represent an immune-driven variant emerging in response to a distinct inflammatory milieu. The lack of *STAT3* mutations further supports the genetic diversity of T-LGLL and underscores the likelihood of alternative molecular drivers.

The association between T-LGLL and B-cell lymphoproliferative disorders, such as hairy cell leukaemia, chronic lymphocytic leukaemia and various lymphomas has been previously reported [10]. The co-occurrence of HL and T-LGLL is extremely rare, with a single multicenter study reporting 2 out of 432 T-LGLL patients with HL coexistence [8]. This suggests an uncommon but possible association since T-LGLL often arises in the context of chronic immune activation, as seen in the inflammatory milieu of HL which is associated with abundant cytokine production and immune cell infiltration, possibly serving as a chronic antigenic stimulus and promoting clonal T-cell expansion [11]. Furthermore, treatment such as chemotherapy and radiation may exert selection pressure, suppressing biclonality and enabling expansion of a resistant clonal population [9]. Due to the retrospective nature of this case report, the proposed rational remains speculative, as direct cytokine profiling or functional immune assays were not performed.

This case highlights the importance of recognizing atypical lymphocyte expansions during lymphoma workup. Clinically, this overlap complicates management. T-LGLL typically follows an indolent course and is often managed conservatively, unless cytopenias or autoimmune complications arise. Priority in treatment must be given to the relapsed HL, as aggressive therapy is essential for disease control. However, immunosuppressive regimens typically used for T-LGLL must, therefore, be balanced against the risks of exacerbating HL or inducing secondary infections. Long-term follow-up with haematologic monitoring is crucial to detect cytopenias or further clonal evolution.

## Data Availability

The data that supports this report are not publicly available due to privacy and ethical concerns. However, it can be requested from the corresponding author.
